# Climate Change Worry and Flourishing Among Chinese University Students: The Roles of Anxiety-Depressive Symptoms and Physical Activity

**DOI:** 10.3390/healthcare14121624

**Published:** 2026-06-09

**Authors:** Shiqi Liu, Yanli Tan, Liuhong Zang

**Affiliations:** School of Physical Education, Xinjiang Normal University, Urumqi 830017, China

**Keywords:** climate change worry, flourishing, anxiety and depressive symptoms, physical activity, conditional process analysis, university students

## Abstract

**Background/Objectives:** Climate change worry is an emerging concern in youth mental health, but little is known about how it is associated with positive psychological functioning among university students. This study examined whether climate change worry was associated with flourishing and whether this association showed a cross-sectional statistical indirect effect through anxiety and depressive symptoms, with physical activity specified as a first-stage boundary condition. **Methods:** A cross-sectional anonymous survey was conducted in 2026 using convenience sampling among students from four universities located in three provincial-level regions of China, covering southern, western, and central areas. After predefined quality control procedures, 2826 valid responses were included. Climate change worry, anxiety and depressive symptoms, flourishing, and physical activity were assessed using the Climate Change Worry Scale (CCWS), the Patient Health Questionnaire-4 (PHQ-4), the Flourishing Scale (FS), and the Physical Activity Rating Scale-3 (PARS-3), respectively. Pearson correlations and conditional process analyses were conducted using the PROCESS macro, with 5000 bootstrap samples. **Results:** Climate change worry was positively associated with anxiety and depressive symptoms (r = 0.331, *p* < 0.001) and negatively associated with flourishing (r = −0.193, *p* < 0.001). Anxiety and depressive symptoms were negatively associated with flourishing (r = −0.486, *p* < 0.001). The cross-sectional statistical indirect effect through anxiety and depressive symptoms was significant (indirect effect = −0.1277, 95% bootstrap CI: [−0.1441, −0.1123]). Physical activity was statistically associated with a weaker first-stage association between climate change worry and anxiety/depressive symptoms (B = −0.0014, *p* < 0.001; ΔR^2^ = 0.0064). The index of moderated mediation was significant (0.0014, 95% bootstrap CI: [0.0008, 0.0020]). **Conclusions:** Climate change worry was statistically associated with lower flourishing, primarily through higher anxiety and depressive symptoms. Physical activity was associated with a weaker first-stage association, but the moderation effect was small in practical magnitude. Given the cross-sectional and self-report design, these findings should be interpreted as conditional statistical associations rather than causal or protective effects.

## 1. Introduction

Global climate change has increasingly moved from an environmental science issue to a public health and mental health concern. Extreme weather, ecological degradation and uncertainty about future living conditions may shape young people’s expectations of safety, development and wellbeing [1]. The health risks associated with climate change are not limited to physical disease. The Lancet Countdown has emphasized that climate-related health threats are intensifying and may affect population wellbeing across the life course [2]. Within psychology, climate change can be understood as a chronic and macrosocial stressor that is difficult for individuals to control directly. Unlike many daily stressors, it is future-oriented, uncertain and often experienced through repeated exposure to information about environmental risk [3]. Reviews of climate change and mental health have linked climate-related exposure and concern with anxiety, depression, stress, sleep disturbance and other mental health indicators. At the same time, this literature remains heterogeneous in terms of measurement tools, study populations and cultural settings [4]. From a public health perspective, climate-related psychological responses should not be automatically equated with clinical disorder. However, persistent worry, helplessness and rumination may become linked with more general psychological distress when individuals perceive climate threats as uncontrollable and personally relevant [5]. A recent systematic review reported positive associations between eco-anxiety and psychological distress, anxiety symptoms, depressive symptoms and stress symptoms. This provides an empirical basis for examining how climate-related worry may connect with common affective symptoms among students [6].

Conceptually, climate change anxiety and climate change worry overlap but are not identical. Climate anxiety usually refers to anxiety-related responses and possible functional impairment, whereas climate change worry focuses more narrowly on repetitive, future-oriented concern about climate change [7]. This distinction is important because the present study used the Climate Change Worry Scale rather than a climate anxiety scale. Accordingly, the manuscript uses the term climate change worry when referring to the focal construct, and uses climate anxiety only when describing studies that specifically measured anxiety-oriented climate responses. Evidence from young people and university students suggests that climate-related emotions may be associated with both distress and pro-environmental engagement, indicating that worry should not be automatically interpreted as a clinical disorder [8,9,10,11,12,13]. In the Chinese university context, empirical evidence on climate-related worry and positive psychological functioning remains limited, which supports the need to examine this issue in the present sociocultural setting.

Climate change worry may be relevant not only to symptom levels, but also to positive psychological functioning. Repeated worry, perceived helplessness and future uncertainty may coincide with lower meaning, optimism and engagement in everyday life [14,15,16]. Flourishing is a core indicator of positive mental health that reflects meaning, supportive relationships, competence, optimism and social contribution [17,18,19]. The simplified Chinese version of the Flourishing Scale has shown acceptable psychometric properties, supporting its use in Chinese student samples [20]. Therefore, flourishing was selected as the positive mental health outcome in the present study.

The present study assessed anxiety and depressive symptoms using the Patient Health Questionnaire-4 (PHQ-4). The PHQ-4 is an ultra-brief screening instrument that combines two anxiety items and two depressive items and is suitable for large-scale survey research [21,22]. In this study, the PHQ-4 total score was interpreted as a brief indicator of anxiety and depressive symptom burden rather than as a diagnostic measure of clinical anxiety or depression [23,24].

Physical activity was examined as a modifiable behavioural factor that may operate as a statistical boundary condition in the association between climate change worry and affective symptoms. International guidelines and reviews indicate that physical activity is relevant to mental health, stress regulation and wellbeing [25,26,27,28,29,30,31,32]. However, given the cross-sectional design of the present study, physical activity is interpreted here as a variable associated with weaker or stronger statistical associations rather than as evidence of a causal protective effect.

Theoretically, the present study primarily drew on conservation of resources theory and stress appraisal theory. Conservation of resources theory proposes that stress is more likely when valued resources are threatened, lost or insufficiently replenished [33,34]. Stress appraisal theory suggests that emotional responses intensify when a threat is appraised as serious and difficult to control [35,36]. From this perspective, climate change worry may reflect perceived threat and resource strain, whereas physical activity may represent a behavioural resource related to stress regulation [37,38,39]. Flourishing was treated as the positive psychological functioning outcome, consistent with broader positive mental health perspectives [40,41].

On this basis, the present study constructed a cross-sectional conditional process model. We hypothesized that climate change worry would be negatively associated with flourishing (H1), that this association would show a cross-sectional statistical indirect effect through anxiety and depressive symptoms (H2), that physical activity would be statistically associated with a weaker first-stage association between climate change worry and anxiety/depressive symptoms (H3), and that the statistical indirect association would vary across levels of physical activity (H4).

## 2. Materials and Methods

### 2.1. Study Design and Participants

This cross-sectional study was conducted in 2026 using an anonymous questionnaire survey design. Participants were recruited from four universities located in three provincial-level regions of China, covering southern, western and central areas. Anonymous questionnaires were distributed using convenience sampling, with recruitment conducted primarily through university-based networks and online survey links. The survey link was distributed by trained members of the research team through university-based networks, including class groups, student communication groups and teacher-assisted dissemination channels. Participants accessed the questionnaire through an online survey platform. Before completing the questionnaire, they read an informed consent statement explaining the study purpose, voluntary participation, anonymity and the right to withdraw. Only participants who provided electronic informed consent could proceed to the questionnaire.

Participants were eligible if they were enrolled university students, were aged 18 years or older, could understand and complete the questionnaire independently, and provided informed consent. To improve data quality, several predefined screening procedures were applied before analysis. Responses were excluded if participants did not complete the core scales required to calculate the CCWS, PHQ-4, or FS total scores, failed the embedded attention-check item, completed the entire questionnaire in less than 120 s, showed obvious patterned responding, or provided logically inconsistent demographic information.

The attention-check item was embedded in the flourishing section and instructed participants to select “Agree” (score = 6); this item was not included in the calculation of the FS total score. Patterned responding was defined as selecting the same response option for 15 or more consecutive core-scale items or showing a clear mechanical response pattern. The 120-s threshold was used as a pragmatic indicator of insufficient engagement because the questionnaire contained multiple psychological scales and demographic items that required careful reading. These criteria were used to identify responses unlikely to reflect attentive participation rather than to make absolute judgments about individual respondents. Of the 3063 responses initially collected, 237 were excluded according to these criteria, leaving 2826 valid responses for analysis, with an effective response rate of 92.26%.

The study was reviewed and approved by the Ethics Committee of Xinjiang Normal University before data collection (ethics review number: XJNU2026LLSC52). All participants provided informed consent before completing the questionnaire, and no personally identifying information was collected.

### 2.2. Measures

Climate change worry was measured using the CCWS. A fully validated Chinese version of the CCWS was not available for the present population. Therefore, the scale was translated using a translation-back-translation procedure before the main survey. A cognitive pilot test with 39 undergraduates was used to check item clarity, and an independent pilot sample of 62 students completed the revised Chinese CCWS, yielding a Cronbach’s alpha of 0.84. These steps supported only preliminary linguistic clarity and internal consistency; they should not be interpreted as a full psychometric validation of the Chinese CCWS. Future studies should further examine its factor structure, measurement invariance, convergent validity, discriminant validity and test–retest reliability in Chinese samples.

The CCWS contains 10 items, which are rated from 1 (never) to 5 (always). In the present study, the total score was used, with higher scores indicating higher climate change worry.

Anxiety and depressive symptoms were assessed with the PHQ-4. The four items are rated from 0 (not at all) to 3 (nearly every day), and the total score ranges from 0 to 12, with higher scores indicating greater anxiety and depressive symptom burden. The PHQ-4 was used as a brief screening measure rather than as a diagnostic instrument for clinical anxiety or depressive disorders.

Flourishing was assessed with the Flourishing Scale. The scale consists of eight substantive items that are rated from 1 (strongly disagree) to 7 (strongly agree), with higher total scores reflecting higher flourishing. An additional attention-check item was embedded in this section and instructed participants to select “Agree” (score = 6); this item was not included in the calculation of the FS total score.

Physical activity was assessed with the Physical Activity Rating Scale-3 (PARS-3). The scale assesses exercise intensity, duration and frequency. Because the duration item was coded from 1 to 5 in the dataset, the physical activity score was calculated as intensity × (duration − 1) × frequency, yielding scores ranging from 0 to 100. Higher scores indicate greater physical activity. Because PARS-3 is based on self-report and uses a multiplicative scoring formula, the resulting score may be affected by recall bias, social desirability bias and relatively high distributional variability.

Demographic covariates included sex, age, grade, family residence, only-child status and exposure to climate change information. Exposure to climate change information was assessed using a single self-report item asking how frequently participants had been exposed to climate change information in daily life or through media. Responses were rated on a five-point scale: 1 = almost none, 2 = low, 3 = moderate, 4 = high and 5 = very high. Only-child status was included as a demographic covariate because it may reflect differences in family structure and perceived family resource availability in the Chinese sociocultural context, which may be relevant to student psychological adjustment.

### 2.3. Statistical Analysis

Data were analysed using IBM SPSS Statistics version 26.0 and the PROCESS macro version 4.2. PROCESS Model 4 was used to examine the cross-sectional statistical indirect effect through anxiety and depressive symptoms, and PROCESS Model 7 was used to examine whether physical activity was associated with a weaker first-stage association [42]. Model 7 was selected because the study focused on whether physical activity functioned as a first-stage boundary condition in the association between climate change worry and anxiety/depressive symptoms. Conceptually, physical activity was considered a stress-regulation-related behavioural resource that may be more directly relevant to the translation of worry into affective symptoms than to the subsequent association between symptoms and flourishing. Therefore, the moderation term was specified on the first-stage association. Conversely, physical activity was not specified as a second-stage moderator because the association between anxiety/depressive symptoms and flourishing may be more closely related to broader psychosocial resources and overall mental health status than to the immediate stress-regulation role of physical activity.

Indirect effects were evaluated using 5000 bootstrap samples. Effects were considered statistically significant when the 95% bootstrap confidence interval did not include zero [43].

Before constructing the interaction term in the conditional process model, climate change worry and physical activity were mean-centred. This procedure follows standard recommendations for interpreting regression interactions [44].

The index of moderated mediation was used to evaluate whether the statistical indirect association differed across levels of physical activity [45]. Common method bias was examined using Harman’s single-factor test [46]. Because Harman’s single-factor test has limited sensitivity and cannot rule out common method variance on its own, it was treated as a preliminary diagnostic rather than as definitive evidence that common method bias was absent. Regression assumptions and robustness were additionally considered through checks of multicollinearity, residual patterns, heteroscedasticity and potentially influential observations.

Effect sizes and model interpretation were considered alongside statistical significance because small effects can become significant in large samples [47]. The final sample size of 2826 also provided adequate precision for detecting small correlation and regression effects in behavioural research [48].

Skewness and kurtosis were inspected before regression analyses because severe non-normality may affect parametric procedures [49]. Distributional indicators were interpreted according to conventional multivariate statistics guidance [50].

The reporting of this observational study followed the STROBE framework where applicable [51]. Because the study was cross-sectional, indirect and conditional process results were interpreted as statistical associations rather than evidence of temporal or causal ordering [52].

Longitudinal designs are needed to test mediation models in a way that better addresses temporal sequence [53]. Methodological work on mediation analysis also indicates that indirect effects require careful interpretation when data are nonexperimental [54].

Bootstrap procedures improve estimation of indirect effects, but they do not remove design-based limitations such as omitted variables, reciprocal effects or measurement error [55].

## 3. Results

Harman’s single-factor test was conducted using the core scale items. The first unrotated factor explained 24.44% of the variance, which was below the commonly used 40% threshold. This result suggested that a dominant single factor was not evident; however, it was interpreted only as a preliminary check because Harman’s test cannot definitively rule out common method variance. Additional regression diagnostics did not suggest serious violations in the main regression models. Across the total-effect, mediator, outcome and first-stage moderation models, variance inflation factors ranged from 1.002 to 3.597, indicating no serious multicollinearity. Breusch-Pagan tests did not indicate evident heteroscedasticity (all *p* ≥ 0.204). Cook’s distance values were small in all models (maximum = 0.0103, with no value > 1.0), suggesting that no single observation exerted excessive influence on the regression estimates.

The demographic characteristics of the participants are presented in Table 1. Among the 2826 participants, 1346 were male (47.63%) and 1480 were female (52.37%). The mean age was 20.45 years (SD = 1.71), with an age range of 18 to 28 years.

Reliability and descriptive statistics are shown in Table 2. Cronbach’s alpha values were 0.885 for the CCWS, 0.745 for the PHQ-4 and 0.835 for the Flourishing Scale. PARS-3 was treated as a composite activity score calculated using a formula rather than as a reflective psychological scale.

All variables showed acceptable distributional characteristics. Absolute skewness values were below 2 and absolute kurtosis values were below 7, supporting the use of parametric correlation and regression analyses.

Pearson correlations are shown in Table 3. Climate change worry was positively correlated with anxiety and depressive symptoms (r = 0.331, *p* < 0.001) and negatively correlated with flourishing (r = −0.193, *p* < 0.001). Anxiety and depressive symptoms were negatively correlated with flourishing (r = −0.486, *p* < 0.001). Physical activity was negatively correlated with anxiety and depressive symptoms (r = −0.102, *p* < 0.001) and positively correlated with flourishing (r = 0.069, *p* < 0.001), but it was not significantly correlated with climate change worry (r = −0.029, *p* = 0.117). Although several correlations reached statistical significance, they were small in magnitude. In particular, the correlation between physical activity and flourishing was very small, suggesting limited practical relevance despite statistical significance in this large sample.

The cross-sectional statistical indirect effect results are presented in Table 4 and Table 5. The total association between climate change worry and flourishing was significant (B = −0.1573, β = −0.192, *p* < 0.001). Climate change worry was positively associated with anxiety and depressive symptoms (B = 0.1283, β = 0.329, *p* < 0.001), and anxiety and depressive symptoms were negatively associated with flourishing (B = −0.9950, β = −0.473, *p* < 0.001). After anxiety and depressive symptoms were included in the model, the direct association between climate change worry and flourishing remained statistically significant but weak (B = −0.0296, β = −0.036, *p* = 0.038).

The statistical indirect effect of climate change worry on flourishing through anxiety and depressive symptoms was significant (B = −0.1277, 95% bootstrap CI: [−0.1441, −0.1123]). This indirect association represented 81.16% of the total statistical association. However, this proportion should be interpreted only as a decomposition of cross-sectional associations rather than as evidence of causal explanation.

The conditional process results are shown in Table 6 and Table 7. In the first-stage model predicting anxiety and depressive symptoms, climate change worry was positively associated with anxiety and depressive symptoms (B = 0.1264, β = 0.324, *p* < 0.001), whereas physical activity was negatively associated with anxiety and depressive symptoms (B = −0.0115, β = −0.094, *p* < 0.001). The interaction between climate change worry and physical activity was significant (B = −0.0014, β = −0.080, *p* < 0.001), with an additional explained variance of ΔR^2^ = 0.0064. This increase in explained variance was small, indicating limited practical magnitude.

**Figure 1:**
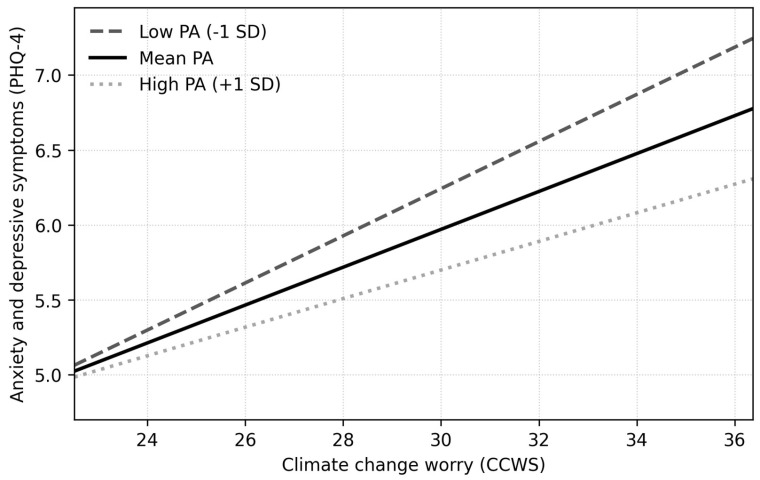
Simple slopes for the association between climate change worry and anxiety and depressive symptoms in students with low, mean and high levels of physical activity. Low and high physical activity represent values one standard deviation below and above the mean of PARS-3, respectively.

Simple slope analysis showed that climate change worry was positively associated with anxiety and depressive symptoms in students with low, mean and high levels of physical activity. However, the slope was stronger for low physical activity (B = 0.1581, *p* < 0.001) than for high physical activity (B = 0.0946, *p* < 0.001), as illustrated in Figure 1. This result indicates a conditional association pattern rather than evidence that physical activity causally protects students from climate-related distress.

Conditional indirect effects also decreased as physical activity increased. The indirect effect was −0.1573 for low physical activity, −0.1257 for mean physical activity and −0.0941 for high physical activity. The index of moderated mediation was significant (0.0014, 95% bootstrap CI: [0.0008, 0.0020]), supporting the conditional statistical association hypothesis. Nevertheless, the small interaction ΔR^2^ indicates that the practical magnitude of this moderation should be interpreted cautiously.

## 4. Discussion

The present study examined the association between climate change worry and flourishing among university students in China using a cross-sectional conditional process framework. Climate change worry was associated with higher anxiety and depressive symptoms and lower flourishing. The association between climate change worry and flourishing showed a significant cross-sectional statistical indirect effect through anxiety and depressive symptoms, and physical activity was related to a weaker first-stage association between climate change worry and these symptoms. These findings should be interpreted as conditional statistical associations rather than as evidence of causal mechanisms. The result is consistent with recent student-based conditional process studies in which psychological symptoms were statistically positioned between risk factors and behavioural or wellbeing outcomes [56,57,58]. The direct association between climate change worry and flourishing remained statistically significant after anxiety and depressive symptoms were included, but the magnitude was very small. This suggests that other unmeasured factors, such as ecological helplessness, future uncertainty, perceived ineffectiveness of climate action or changes in life meaning, may also be relevant.

The first-stage conditional association indicated that higher physical activity was related to a weaker positive association between climate change worry and anxiety and depressive symptoms. Similar uses of physical activity as a conditional factor have been reported in student research on bullying victimization, mobile phone addiction and sleep quality [59,60]. However, the present effect size was small. The interaction term explained only 0.64% additional variance. Therefore, this result should not be taken to mean that physical activity strongly protects students from climate-related distress, nor should it be interpreted as therapeutic or intervention evidence. A more cautious reading is that physical activity may be a modest behavioural factor under which the worry-symptom association differs slightly. Research directly linking physical activity with climate-related worry or eco-anxiety remains limited, and this finding requires replication in longitudinal and intervention studies [61,62].

The study contributes to the climate mental health literature in three ways. First, it focuses on climate change worry rather than only climate anxiety, which helps distinguish future-oriented concern from more impairment-focused constructs. Second, it examines flourishing as a positive psychological outcome. Reviews of climate concerns and negative emotions in young people have called for broader outcome models that include both distress and wellbeing [63]. Third, the study integrates a behavioural factor into the model while explicitly recognizing that the observed physical activity moderation was small in magnitude [64].

Despite these contributions, the findings must be interpreted considering several limitations. First, the cross-sectional data cannot determine whether climate change worry preceded anxiety and depressive symptoms or whether students with higher symptoms were more likely to report climate change worry. Reverse or bidirectional associations are plausible. Second, the study relied on convenience sampling through online links and university networks, which may have introduced self-selection bias. Third, although participants were recruited from four universities located in three provincial-level regions covering southern, western and central China, the geographical and institutional coverage remained limited; therefore, the findings should be generalized to all Chinese university students with caution. Specific university names, institutional types, academic majors and direct socioeconomic indicators, such as household income and parental education, were not collected, limiting a more detailed evaluation of sample representativeness. Fourth, the CCWS translation and pilot testing supported preliminary use in this sample but did not constitute a full validation of a Chinese CCWS; future research should conduct confirmatory factor analysis, convergent and discriminant validity testing, measurement invariance analyses and test–retest reliability assessment. Fifth, anxiety and depressive symptoms were assessed using the PHQ-4 total score. Although this score is useful as a brief indicator of overall psychological distress, it may obscure differences between anxiety and depression. Because climate change worry is conceptually closer to anxiety than to depression, future studies should examine GAD-2 and PHQ-2 subscales separately, or use more comprehensive anxiety- and depression-specific instruments. Sixth, PARS-3 was based on self-report and may be affected by recall bias, social desirability bias and score variability from the multiplicative formula. Finally, the use of Harman’s single-factor test was only a preliminary check of common method bias, and unmeasured variables such as neuroticism, trait anxiety, pre-existing mental health conditions, coping style and social support may confound the observed associations.

Future research can consider objective or contextual climate exposures, longitudinal designs and objective physical activity measures. Large-scale work has shown that climate and weather conditions may be associated with mental health risks, suggesting that subjective worry and objective exposure should be examined together [65]. More comprehensive models may further integrate climate worry, coping, social support, hope and climate action [66,67,68]. Longitudinal and experimental designs are especially needed to determine whether the statistical associations observed here reflect temporal processes, reciprocal relationships or shared underlying vulnerabilities. Future studies should also examine whether these associations differ by sex, age, and level of exposure to climate change information because these variables may shape students’ climate-related psychological responses and wellbeing.

## 5. Conclusions

Climate change worry was associated with lower flourishing among university students, and this association showed a cross-sectional statistical indirect effect through anxiety and depressive symptoms. Physical activity was related to a weaker first-stage association between climate change worry and anxiety/depressive symptoms, but the moderation effect was small in practical magnitude. These findings point to anxiety and depressive symptoms as important psychological correlates and to physical activity as a modest behavioural condition. Because the study used a cross-sectional self-report design, the results should not be interpreted as causal or as evidence that physical activity has a strong protective or therapeutic effect.

## Figures and Tables

**Table 1:** Demographic characteristics of participants (N = 2826).

Characteristic	n/M	%/SD
**Sex**		
Male	1346	47.63
Female	1480	52.37
Age, years	20.45	1.71
Age range	18–28	
**Grade**		
First year	857	30.33
Second year	771	27.28
Third year	625	22.12
Fourth year	433	15.32
Postgraduate or above	140	4.95
**Only-child status**		
Only child	1304	46.14
Not only child	1522	53.86
**Family residence**		
Urban	1531	54.18
Rural	1295	45.82
**Exposure to climate change information**		
Almost none	225	7.96
Low	630	22.29
Moderate	1089	38.54
High	650	23.00
Very high	232	8.21

Note. M and SD are reported for age; n and percentages are reported for categorical variables.

**Table 2:** Descriptive statistics, distribution indicators and reliability.

Variable	M	SD	Min	Max	Skewness	Kurtosis	Cronbach’s Alpha
CCWS	29.44	6.93	11	49	0.02	−0.30	0.885
PHQ-4	5.90	2.70	0	12	0.04	−0.55	0.745
FS	31.80	5.67	10	51	−0.02	−0.03	0.835
PARS-3	27.97	22.09	0	100	1.11	0.93	-

Note. CCWS = Climate Change Worry Scale; PHQ-4 = Patient Health Questionnaire-4; FS = Flourishing Scale; PARS-3 = Physical Activity Rating Scale-3. PARS-3 was calculated as a composite physical activity score and was not primarily evaluated using internal consistency.

**Table 3:** Pearson correlations among the main variables.

Variable	1	2	3	4
1. CCWS	-			
2. PHQ-4	0.331 ***	-		
3. FS	−0.193 ***	−0.486 ***	-	
4. PARS-3	−0.029	−0.102 ***	0.069 ***	-

Note. *** *p* < 0.001.

**Table 4:** Regression results for the statistical indirect association model.

Path	Outcome	Predictor	B	SE	β	t	95% CI
Total effect c	FS	CCWS	−0.1573	0.0151	−0.192	−10.396 ***	[−0.1870, −0.1276]
a path	PHQ-4	CCWS	0.1283	0.0069	0.329	18.557 ***	[0.1148, 0.1419]
Direct effect c’	FS	CCWS	−0.0296	0.0143	−0.036	−2.075 *	[−0.0576, −0.0016]
b path	FS	PHQ-4	−0.9950	0.0367	−0.473	−27.095 ***	[−1.0670, −0.9230]

Note. Models controlled for sex, age, grade, family residence, only-child status and exposure to climate change information. * *p* < 0.05, *** *p* < 0.001. β = standardized coefficients.

**Table 5:** Bootstrap estimates of total, direct and indirect associations.

Effect	B	Boot SE	95% Bootstrap CI	Proportion
Total effect	−0.1573	0.0153	[−0.1875, −0.1275]	-
Direct effect	−0.0296	0.0144	[−0.0578, −0.0015]	18.84%
Indirect effect	−0.1277	0.0082	[−0.1441, −0.1123]	81.16%

Note. Bootstrap resampling was conducted using 5000 samples. CI = confidence interval.

**Table 6:** First-stage moderation model predicting anxiety and depressive symptoms.

Predictor	B	SE	β	t	*p*	95% CI
CCWS	0.1264	0.0069	0.324	18.395	<0.001	[0.1129, 0.1398]
PARS-3	−0.0115	0.0022	−0.094	−5.295	<0.001	[−0.0158, −0.0072]
CCWS × PA	−0.0014	0.0003	−0.080	−4.554	<0.001	[−0.0021, −0.0008]

Note. Outcome variable = PHQ-4. The model controlled for sex, age, grade, family residence, only-child status and exposure to climate change information. CCWS and PARS-3 were mean-centred before constructing the interaction term. R^2^ = 0.1289; interaction ΔR^2^ = 0.0064, *p* < 0.001. β = standardized coefficients.

**Table 7:** Simple slopes, conditional indirect effects and the index of moderated mediation.

Analysis	Physical Activity Level	B/Effect	SE/Boot SE	*p*	95% CI
Simple slope	Low (−1 SD)	0.1581	0.0096	<0.001	[0.1392, 0.1770]
Simple slope	Mean	0.1264	0.0069	<0.001	[0.1129, 0.1398]
Simple slope	High (+1 SD)	0.0946	0.0099	<0.001	[0.0751, 0.1141]
Indirect effect	Low (−1 SD)	−0.1573	0.0112	-	[−0.1798, −0.1361]
Indirect effect	Mean	−0.1257	0.0080	-	[−0.1418, −0.1105]
Indirect effect	High (+1 SD)	−0.0941	0.0100	-	[−0.1138, −0.0748]
Index of moderated mediation	-	0.0014	0.0003	-	[0.0008, 0.0020]

Note. Conditional indirect effects and the index were estimated using 5000 bootstrap samples.

## Data Availability

The datasets analysed during the current study are not publicly available due to privacy and ethical restrictions. The data were collected from human participants and include demographic and psychological health-related information. The informed consent approved for this study did not include permission for unrestricted public data sharing. De-identified data supporting the findings of this study may be made available from the corresponding author upon reasonable request, subject to approval by the institutional ethics committee and compliance with relevant privacy and data protection requirements.

## References

[1] IPCC (2023). Climate Change 2023: Synthesis Report. Contribution of Working Groups I, II and III to the Sixth Assessment Report of the Intergovernmental Panel on Climate Change.

[2] Romanello M., Walawender M., Hsu S.C., Moskeland A., Palmeiro-Silva Y., Scamman D., Ali Z., Ameli N., Angelova D., Ayeb-Karlsson S. (2024). The 2024 report of the Lancet Countdown on health and climate change: Facing record-breaking threats from delayed action. Lancet.

[3] Palinkas L.A., Wong M. (2020). Global climate change and mental health. Curr. Opin. Psychol..

[4] Cianconi P., Betro S., Janiri L. (2020). The impact of climate change on mental health: A systematic descriptive review. Front. Psychiatry.

[5] Charlson F., Ali S., Benmarhnia T., Pearl M., Massazza A., Augustinavicius J., Scott J.G. (2021). Climate change and global mental health: A scoping review. Int. J. Environ. Res. Public Health.

[6] Cosh S.M., Ryan R., Fallander K., Robinson K., Tognela J., Tully P.J., Lykins A.D. (2024). The relationship between climate change and mental health: A systematic review of the association between eco-anxiety, psychological distress, and symptoms of major affective disorders. BMC Psychiatry.

[7] Clayton S. (2020). Climate anxiety: Psychological responses to climate change. J. Anxiety Disord..

[8] Hickman C., Marks E., Pihkala P., Clayton S., Lewandowski R.E., Mayall E.E., Wray B., Mellor C., van Susteren L. (2021). Climate anxiety in children and young people and their beliefs about government responses to climate change: A global survey. Lancet Planet. Health.

[9] Ma T., Moore J., Cleary A. (2022). Climate change impacts on the mental health and wellbeing of young people: A scoping review of risk and protective factors. Soc. Sci. Med..

[10] Ogunbode C.A., Doran R., Hanss D., Ojala M., Salmela-Aro K., Broek K.L.v.D., Bhullar N., Aquino S.D., Marot T., Schermer J.A. (2022). Climate anxiety, wellbeing and pro-environmental action: Correlates of negative emotional responses to climate change in 32 countries. J. Environ. Psychol..

[11] Clayton S., Karazsia B.T. (2020). Development and validation of a measure of climate change anxiety. J. Environ. Psychol..

[12] Stewart A.E. (2021). Psychometric properties of the Climate Change Worry Scale. Int. J. Environ. Res. Public Health.

[13] Larionow P., Gawrych M., Mackiewicz J., Michalak M., Mudło-Głagolska K., Preece D.A., Stewart A.E. (2024). The Climate Change Worry Scale and its links with demographics and mental health outcomes in a Polish sample. Healthcare.

[14] Whitmarsh L., Player L., Jiongco A., James M., Williams M., Marks E., Kennedy-Williams P. (2022). Climate anxiety: What predicts it and how is it related to climate action?. J. Environ. Psychol..

[15] Stanley S.K., Hogg T.L., Leviston Z., Walker I. (2021). From anger to action: Differential impacts of eco-anxiety, eco-depression, and eco-anger on climate action and wellbeing. J. Clim. Change Health.

[16] Bouman T., Verschoor M., Albers C.J., Böhm G., Fisher S.D., Poortinga W., Whitmarsh L., Steg L. (2020). When worry about climate change leads to climate action: How values, worry and personal responsibility relate to various climate actions. Glob. Environ. Change.

[17] Keyes C.L.M. (2002). The mental health continuum: From languishing to flourishing in life. J. Health Soc. Behav..

[18] Keyes C.L.M. (2005). Mental illness and/or mental health? Investigating axioms of the complete state model of health. J. Consult. Clin. Psychol..

[19] Diener E., Wirtz D., Tov W., Kim-Prieto C., Choi D.-W., Oishi S., Biswas-Diener R. (2010). New well-being measures: Short scales to assess flourishing and positive and negative feelings. Soc. Indic. Res..

[20] Tang X., Duan W., Wang Z., Liu T. (2016). Psychometric evaluation of the simplified Chinese version of Flourishing Scale. Res. Soc. Work. Pract..

[21] Kroenke K., Spitzer R.L., Williams J.B.W., Lowe B. (2009). An ultra-brief screening scale for anxiety and depression: The PHQ-4. Psychosomatics.

[22] Lowe B., Wahl I., Rose M., Spitzer C., Glaesmer H., Wingenfeld K., Schneider A., Brähler E. (2010). A 4-item measure of depression and anxiety: Validation and standardization of the Patient Health Questionnaire-4 in the general population. J. Affect. Disord..

[23] Spitzer R.L., Kroenke K., Williams J.B.W., Lowe B. (2006). A brief measure for assessing generalized anxiety disorder: The GAD-7. Arch. Intern. Med..

[24] Kroenke K., Spitzer R.L., Williams J.B.W. (2001). The PHQ-9: Validity of a brief depression severity measure. J. Gen. Intern. Med..

[25] Bull F.C., Al-Ansari S.S., Biddle S., Borodulin K., Buman M.P., Cardon G., Carty C., Chaput J.-P., Chastin S., Chou R. (2020). World Health Organization 2020 guidelines on physical activity and sedentary behaviour. Br. J. Sports Med..

[26] World Health Organization (2020). WHO Guidelines on Physical Activity and Sedentary Behaviour.

[27] Schuch F.B., Vancampfort D., Firth J., Rosenbaum S., Ward P.B., Silva E.S., Hallgren M., De Leon A.P., Dunn A.L., Deslandes A.C. (2018). Physical activity and incident depression: A meta-analysis of prospective cohort studies. Am. J. Psychiatry.

[28] Stubbs B., Vancampfort D., Rosenbaum S., Firth J., Cosco T., Veronese N., Salum G.A., Schuch F.B. (2017). An examination of the anxiolytic effects of exercise for people with anxiety and stress-related disorders: A meta-analysis. Psychiatry Res..

[29] Mikkelsen K., Stojanovska L., Polenakovic M., Bosevski M., Apostolopoulos V. (2017). Exercise and mental health. Maturitas.

[30] Mandolesi L., Polverino A., Montuori S., Foti F., Ferraioli G., Sorrentino P., Sorrentino G. (2018). Effects of physical exercise on cognitive functioning and wellbeing: Biological and psychological benefits. Front. Psychol..

[31] Lubans D., Richards J., Hillman C., Faulkner G., Beauchamp M., Nilsson M., Kelly P., Smith J., Raine L., Biddle S. (2016). Physical activity for cognitive and mental health in youth: A systematic review of mechanisms. Pediatrics.

[32] Biddle S.J.H., Ciaccioni S., Thomas G., Vergeer I. (2019). Physical activity and mental health in children and adolescents: An updated review of reviews and an analysis of causality. Psychol. Sport. Exerc..

[33] Hobfoll S.E. (1989). Conservation of resources: A new attempt at conceptualizing stress. Am. Psychol..

[34] Hobfoll S.E., Halbesleben J., Neveu J.P., Westman M. (2018). Conservation of resources in the organizational context: The reality of resources and their consequences. Annu. Rev. Organ. Psychol. Organ. Behav..

[35] Lazarus R.S., Folkman S. (1984). Stress, Appraisal, and Coping.

[36] McEwen B.S. (1998). Protective and damaging effects of stress mediators. N. Engl. J. Med..

[37] Klaperski S., von Dawans B., Heinrichs M., Fuchs R. (2014). Effects of a 12-week endurance training program on the physiological response to psychosocial stress in men: A randomized controlled trial. J. Behav. Med..

[38] Rimmele U., Seiler R., Marti B., Wirtz P.H., Ehlert U., Heinrichs M. (2009). The level of physical activity affects adrenal and cardiovascular reactivity to psychosocial stress. Psychoneuroendocrinology.

[39] Zschucke E., Renneberg B., Dimeo F., Wüstenberg T., Ströhle A. (2015). The stress-buffering effect of acute exercise: Evidence for HPA axis negative feedback. Psychoneuroendocrinology.

[40] Fredrickson B.L. (2001). The role of positive emotions in positive psychology: The broaden-and-build theory of positive emotions. Am. Psychol..

[41] Ryff C.D. (1989). Happiness is everything, or is it? Explorations on the meaning of psychological well-being. J. Pers. Soc. Psychol..

[42] Hayes A.F. (2022). Introduction to Mediation, Moderation, and Conditional Process Analysis: A Regression-Based Approach.

[43] Preacher K.J., Hayes A.F. (2008). Asymptotic and resampling strategies for assessing and comparing indirect effects in multiple mediator models. Behav. Res. Methods.

[44] Aiken L.S., West S.G. (1991). Multiple Regression: Testing and Interpreting Interactions.

[45] Hayes A.F. (2015). An index and test of linear moderated mediation. Multivar. Behav. Res..

[46] Podsakoff P.M., MacKenzie S.B., Lee J.Y., Podsakoff N.P. (2003). Common method biases in behavioral research: A critical review of the literature and recommended remedies. J. Appl. Psychol..

[47] Cohen J. (1988). Statistical Power Analysis for the Behavioral Sciences.

[48] Faul F., Erdfelder E., Buchner A., Lang A.G. (2009). Statistical power analyses using G*Power 3.1: Tests for correlation and regression analyses. Behav. Res. Methods.

[49] Kline R.B. (2016). Principles and Practice of Structural Equation Modeling.

[50] Tabachnick B.G., Fidell L.S. (2019). Using Multivariate Statistics.

[51] Von Elm E., Altman D.G., Egger M., Pocock S.J., Gøtzsche P.C., Vandenbroucke J.P. (2007). The Strengthening the Reporting of Observational Studies in Epidemiology statement: Guidelines for reporting observational studies. PLoS Med..

[52] Maxwell S.E., Cole D.A. (2007). Bias in cross-sectional analyses of longitudinal mediation. Psychol. Methods.

[53] Cole D.A., Maxwell S.E. (2003). Testing mediational models with longitudinal data: Questions and tips in the use of structural equation modeling. J. Abnorm. Psychol..

[54] MacKinnon D.P., Fairchild A.J., Fritz M.S. (2007). Mediation analysis. Annu. Rev. Psychol..

[55] Shrout P.E., Bolger N. (2002). Mediation in experimental and nonexperimental studies: New procedures and recommendations. Psychol. Methods.

[56] Wang J., Tang L., Liu Y., Wu X., Zhou Z., Zhu S. (2025). Physical activity moderates the mediating role of depression between experiential avoidance and Internet addiction. Sci. Rep..

[57] Peng J., Quan Z., Yan C., Dong J., Chen Z., Liu P. (2025). Physical exercise moderated the mediating role of anxiety between experiential avoidance and the teenagers BrainRot. Sci. Rep..

[58] Peng J., Liu Y., Wang X., Yi Z., Xu L., Zhang F. (2025). Physical and emotional abuse with internet addiction and anxiety as a mediator and physical activity as a moderator. Sci. Rep..

[59] Peng J., Wang J., Chen J., Li G., Xiao H., Liu Y., Zhang Q., Wu X., Zhang Y. (2025). Mobile phone addiction was the mediator and physical activity was the moderator between bullying victimization and sleep quality. BMC Public Health.

[60] Peng J., Liu M., Wang Z., Xiang L., Liu Y. (2025). Anxiety and sleep hygiene among college students, a moderated mediating model. BMC Public Health.

[61] Comoutos N., Karamitrou A., Krommidas C., Morres I.D., Violatzi A., Dimitriou E., Hassandra M., Papaioannou A., Theodorakis Y. (2025). Exploring the relationship between physical activity, flood experience, eco-anxiety, and youth well-being in a changing climate. Sci. Rep..

[62] Söder A., Herr R.M., Görig T., Diehl K. (2025). Climate Change Worry in German university students: Determinants and associations with health-related outcomes. Climate.

[63] Ramadan R., Randell A., Lavoie S., Gao C.X., Manrique P.C., Anderson R., McDowell C., Zbukvic I. (2023). Empirical evidence for climate concerns, negative emotions and climate-related mental ill-health in young people: A scoping review. Early Interv. Psychiatry.

[64] Schwartz S.E., Benoit L., Clayton S., Parnes M.F., Swenson L., Lowe S.R. (2023). Climate change anxiety and mental health: Environmental activism as buffer. Curr. Psychol..

[65] Obradovich N., Migliorini R., Paulus M.P., Rahwan I. (2018). Empirical evidence of mental health risks posed by climate change. Proc. Natl. Acad. Sci. USA.

[66] Doherty T.J., Clayton S. (2011). The psychological impacts of global climate change. Am. Psychol..

[67] Ojala M. (2012). How do children cope with global climate change? Coping strategies, engagement, and well-being. J. Environ. Psychol..

[68] Ojala M. (2012). Regulating worry, promoting hope: How do children, adolescents, and young adults cope with climate change?. Int. J. Environ. Sci. Educ..

